# Injectable Cartilage Shaving: An Autologous and Long Lasting Filler Material for Correction of Minor Contour Deformities in Rhinoplasty

**Published:** 2015-07

**Authors:** Ali Manafi, Zahra Sadat Hamedi, Amir Manafi, Afsaneh Rajabiani, Ahmadreza Rajaee, Farzad Manafi

**Affiliations:** 1 Department of Plastic Surgery, Iran University of Medical Sciences, Tehran, Iran; 2 Digestive Oncology Research Center, Tehran University of Medical Sciences, Tehran, Iran; 3 Department of Pathology, Tehran University of Medical Sciences, Tehran, Iran; 4 Department of Otolaryngology, International Branch of Shiraz University of Medical Sciences, Shiraz, Iran

**Keywords:** Injectable, Cartilage, Shaving, Rhinoplasty

## Abstract

**BACKGROUND:**

Filler materials are gaining popularity in nonsurgical rhinoplasty the major advantages are the ability to camouflage the surface deformities, and also the soft and malleable consistency; while the major drawback of the safe fillers such as hyaluronic acid is short durability. In this study, we evaluated the injectable cartilage shaving as an autologous filler material for correction of minor contour deformities in rhinoplasty.

**METHODS:**

Injectable cartilage shaving was used for correction of surface irregularities in primary or secondary rhinoplasty, and long term results of 128 patients were evaluated. The source of cartilage was autologous septum, rib or less frequently, the ear concha. The material was injected with 14 to 18 gauge needles or blunted tip lipofilling cannulas with 1.3-1.7 mm internal diameters. It was performed whether during the septorhinoplasty or as a separate single procedure without elevation of the flap. Success was defined as the long term survival of the graft in the desired site and absence of recurrent deformity or complications such as extrusion, infection or displacement.

**RESULTS:**

Twenty seven males and 101 females underwent the procedure from May 2008 to January 2014. Mean follow up period was 31 (13-58) months. Ninety five percent of patients were satisfied or very satisfied with the results at the last follow up visits and touch up procedure was performed for the unsatisfied patients.

**CONCLUSION:**

Injectable cartilage shaving is a reliable filler to correct and camouflage the surface irregularities, and it is durable and predictable in long term follow ups.

## INTRODUCTION

Long lasting and predictable functional and aesthetic results in a rhinoplasty procedure usually cannot be achieved without usage of various types of grafts. Plenty of graft materials have been introduced in the literature, each having advantages and disadvantages. There is a global consensus that autologous materials are preferred in rhinoplasty. Foreign materials may be proper choices for skeletal augmentation of some specific areas such as cheek or chin, but they may result in disastrous long term complications when grafted to the nasal skeleton.^1^

Cartilages of the septum, ear concha and rib are the most frequent sources of autologous grafts while covering fascia of the temporalis or rectus abdominis muscle, fascia lata, and calvarial or iliac bone are other examples. Most of the grafts are used alone or in combination, to rebuild or augment a specific structure of the nose^2^ and some advanced techniques in manipulation of the tip and other subunits of the nose have been proposed to adjust the structures as precise as possible,^3^ but sometimes the grafts are used for the purpose of camouflage and improvement of surface aesthetic properties.

Most of these camouflaging grafts are used during operation and via classic incisions but correction of minor surface irregularities or adding volume to specific locations of the nose sometimes can be done without elevation of skin flap using filler materials.^4^ Nonsurgical rhinoplasty that is progressively gaining popularity among surgeons is based on this fact.^5^

Autologous cartilage grafts can be used as crushed,^6^ diced^7^ or grated^8^ material to camouflage the surface irregularities with or without fascia, but placement of these grafts must be done through classic incisions and as a part of a septorhinoplasty procedure. We have designed injectable cartilage shaving which is prepared from septal, costal or less frequently conchal cartilage and can be injected via 14 to 18 gauge needles or blunted tip cannulas of lipofilling with 1.3 to 1.7 mm diameter. It can be introduced from skin or mucosal surface directly to the desired location without the need for any incision or elevation of skin flap; therefore, the injection can be performed as an outpatient procedure.

## MATERIALS AND METHODS

A retrospective review was performed on charts of 128 consecutive cases that underwent open or closed septorhinoplasty using injectable cartilage shaving, from May 2008 to January 2014. The study complied with declaration of Helsinki and was approved by the Ethics Committee of the Iran University of Medical Sciences. All patients were provided with written informed consent. 

Both primary and secondary rhinoplasty cases were included and most common indications for usage of this graft was minor surface contour deformities, the problem was addressed with incremental injection of the cartilage shaving to the desired location with repeated visual and tactile evaluation after each injection.

There were no tools to precisely quantify the outcome. Final evaluation of the long term results was done by the surgeon using visual and tactile findings. Possible complications such as infection, extrusion, displacement, resorption and overcorrection were checked at all follow up visits. Long term survival of the graft in the desired site and absence of recurrent deformity were considered as determinants of success. 

A questionnaire was also given to the patients to evaluate the degree of their satisfaction from the general result of rhinoplasty at least one year after their operation. They were not asked to identify themselves and the questionnaire was collected in a specific folder in the office that contained same questionnaire sheets from other patients. Patients voluntarily chose one of the three choices (very satisfied, satisfied or unhappy with the result) in the questionnaire. Standard photography for rhinoplasty was performed in frontal, lateral, oblique and basal views before operation and at the follow up sessions of 6 months, one year and then each year after operation. Only patients who attended at a minimum of one year follow up were included. 

Sources of the autologous cartilage grafts in this study were nasal septum, rib, or less frequently ear concha. In some cases we would use the excess amounts of cartilage which was implanted in the patient’s own body at the end of previous operations. These grafts were more commonly implanted via the incision for harvesting rib cartilage, but it could be also implanted beneath the scalp or behind the ear pinna, in cases of harvesting temporalis fascia or conchal cartilage.

For preparation of the injectable cartilage shaving, any soft tissue attachments were debrided from the cartilage block. It was firmly fixated with the cartilage forceps in the non-dominant hand of the surgeon, a surgical blade (number 10, 11, or 15) was used to scratch the edges of the cartilage block gently and shave very thin and small pieces. No visible separate piece of cartilage could be identified and the produced material rather resembled as powder (the procedure is described in details in a video file which can be downloaded from the website of the journal). 

Septal and costal cartilages were considered more appropriate in comparison to conchal cartilage which was more pliable and fragile. The material was then soaked in solution of gentamycine in sterile saline and introduced to one or more 1 ml Luer-lock syringes. The effort to find the best location for injection was done using visual and tactile evaluation of the surface and if performed as a part of open rhinoplasty, it was postponed until the skin flap was returned back and sutured at the end of the operation.

This graft material was actually applicable in every part of the nose including radix, dorsum, supratip area, nasal tip, soft triangle, columella, alar base, premaxilla, medial canthus proximities, and at the sides of the piriform aperture. The injection was usually performed at the level of hypodermis to ensure placement of the graft in a fixed location, eliminating the possibility of future displacement. 

In more details, it was used to augment the radix and soften the acute nasofrontal angle, fill the irregular defects of the nasal dorsum, soften the accentuated supratip break, increase the columellar show, increase the columellar labial angle by augmenting the premaxilla, restore enough volume to the dimplings around piriform area, fill the depressions on the surface of collapsed nasal bones adjacent to medial canthus, and restore a diverge appearance at the location of deficient foot plates in base of columella.

The graft material could be introduced without vigorous force through 14 to 18 gauge needles or blunted tip lipofilling cannulas with 1.3-1.7 mm internal diameters, and the process of injection was incremental and cautious. Repeated visual and tactile evaluation was performed until adequate volume was injected. Final molding of the injected material could be done with gentle massage of the overlying skin if it was not injected into a tight pocket.

There was a possibility of embolization of the injected material into the ophthalmic artery when injection was performed in dorsal nasal and periorbital area. This risk would be minimal due to the vasoconstrictor effect of adrenaline, injected before placement of the graft and the possibility would be much less if blunted tip cannulas were used instead of sharp needles. We also performed a histological examination on the fresh samples of cartilage shaving to evaluate the gross structural properties. On the other hand we crushed pieces of the same cartilage blocks to the extent that it could be injected through 14-18 gauge needles and histological examination was performed on the fresh samples to determine the differences with cartilage shaving.

## RESULTS

Age of patients ranged from 17 to 63 years with a mean of 25. Injectable cartilage shaving was used to correct the deformities in decreasing order of frequency in the radix, dorsum, supratip, lateral alar walls, nasal bone surfaces adjacent to medial canthus, alar sill, medial crural foot plates and other locations. Injectable cartilage shaving was prepared from septal cartilage in 96 (75%), from costa in 26 (20%), and from ear concha in 6 (5%) patients. It was used as the only cartilage graft in 19 (15%) and as an adjunction to other block cartilage grafts in 109 (85%) cases. Follow up ranged from 13 to 58 months with an average of 31 months.

**Fig. 1:**
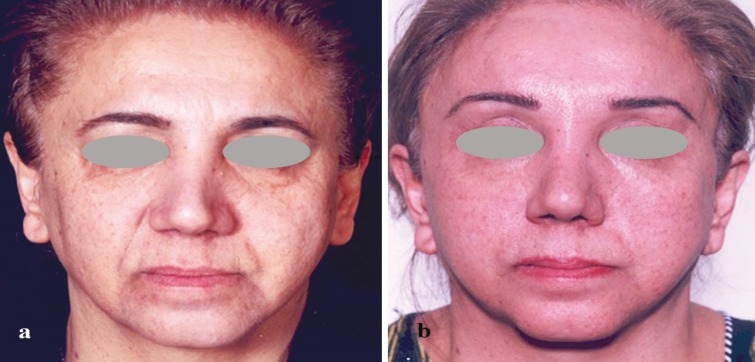
a: A 56 year old lady presented with severe asymmetry of the tip and alar cartilages, and aging face, b: facial rejuvenation and shaved cartilage injection to left side of nose, 3 years post operative feature

**Fig. 2:**
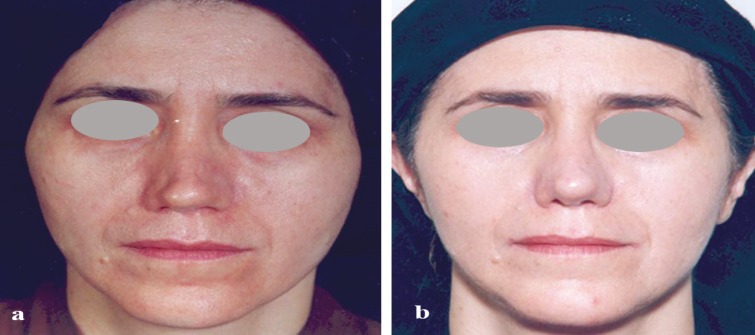
a: A 45 year old lady presented with right side tip bossa formation, tip asymmetry and bilateral alar pinching were the most prominent complaints. b: Postoperative results after 30 months. She has had shaved cartilage injection to defective parts of nose

**Fig. 3:**
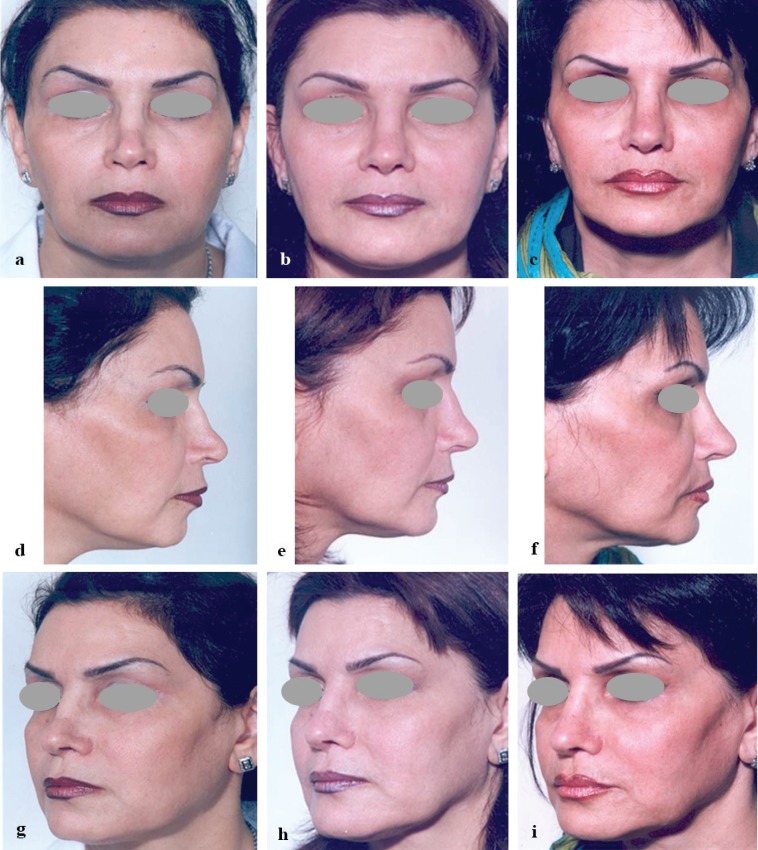
a,d,g: A 48 year old lady with inverted V deformity, bilateral alar pinching and collapse of the internal valve leading to nasal airway obstruction. b,e,h: after secondary rhinoplasty using bilateral spreader graft, bilateral lateral crural strut graft, onlay tip graft (and chin augmentation). c,f,i: 18 months after injection of the cartilage shaving in supratip and radix

The questionnaire was given to the patients at one year or more after operation and general satisfaction was evaluated. Ninety five patients completed the questionnaire. From those 42 (44%) respondents marked very satisfied, 48 (51%) respondents marked satisfied and 5 (5%) respondents marked unhappy with the results. No cases of infection, extrusion, displacement or excess bulging occurred during the course of follow up. None of the patients experienced complete resorption and recurrence of preexisting deformity but partial resorption was seen in almost all of patients to small extents and this was the leading cause of performing revision surgery in 7 (5%) cases. Some of the long term results of injectable cartilage shaving are presented in figures 1-3. (Video)

Figure 1a shows a 56 year old lady presented with severe asymmetry of the tip and alar cartilages, and aging face. She underwent facelift, fat grafting to midface, upper lid blepharoplasty and mastopexy. She had previously undergone two rhinoplasty procedures 5 and 7 years before and refused a comprehensive secondary rhinoplasty. Rib cartilage was harvested through the incision site for mastopexy, and cartilage shaving was prepared and injected into deficient areas of the nose. Figure 1.b demonstrates postoperative results 3 years after injection.

In Figure 2.a, a 45 year old lady was presented with history of two previous rhinoplasty procedures 3 and 4 years ago and also a history of mastopexy. Right side tip bossa formation, tip asymmetry and bilateral alar pinching were the most prominent complaints. Rib cartilage was harvested through the incision of mastopexy. Cartilage shaving was prepared and injected to camouflage the surface deformities. Figure 2.b presents postoperative results after 30 months. 

Figures 3.a,d,g exhibit a 48 year old lady undergone septo rhinoplasty 2 years before and presented with inverted V deformity, bilateral alar pinching and collapse of the internal valve leading to nasal airway obstruction. Figures 3.b,e,h denote to post-secondary rhinoplasty using bilateral spreader graft, bilateral lateral crural strut graft, onlay tip graft (and chin augmentation). The complaints of the patient were addressed except for the mild supratip dimpling. We had reimplanted a piece of rib cartilage beneath the incision of the chest wall and used it again to produce injectable cartilage shaving. Supratip dimpling was filled with precise injection. Figures 3.c,f,i indicate 18 months after injection of the cartilage shaving in supratip.

**Fig. 4:**
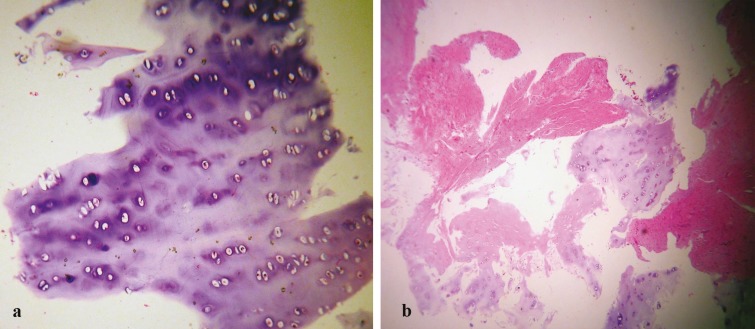
a: Shaved cartilagenous tissue fragments with preserved lacunae, including chondrocytes, associated with normal architecture and usual staining of chondroid matrix. b: Crushed cartilage tissue fragments, with damaged lacunae, some of them devoid of chondrocytes, associated with damaged and degenerated cartilagenous matrix with some induced tearing

Figure 4.a reflect shaved cartilagenous tissue fragments with preserved lacunae, including chondrocytes, associated with normal architecture and usual staining of chondroid matrix. Figure 4.b shows crushed cartilage tissue fragments, with damaged lacunae, some of them devoid of chondrocytes, associated with damaged and degenerated cartilagenous matrix with some induced tearing. We found some gross structural differences between the two groups supratip dimpling (Figure 4). 

## DISCUSSION

Minor contour deformities of the nasal surface may be considered trifle, but they are sometimes the only factors preventing us to achieve perfection. These contour deformities may be a result of trauma, various dermatologic or systemic disorders and previous aesthetic nasal surgeries.^9^ More commonly, at the end of the rhinoplasty procedure, when major adjustments of osteo-cartilagenous skeleton have been achieved and the surgeon redrapes the skin envelope, there will be often small remaining irregularities. Correction of these irregularities using common graft materials are sometimes challenging. Cartilage blocks will usually result in visible and palpable edges if placed as onlay grafts (especially in thin skin patients), Crushed cartilage will have unpredictable course of resorption considering the extent of crushing and fascia or other autologous soft tissue materials need a separate location of harvesting.

The other issue which is usually overlooked is that using malleable materials as onlay grafts without suture fixation during an open rhinoplasty procedure carries the risk of displacement when the skin flap is returned back. Besides, the efficacy of the graft in complete resolution of contour deformity cannot be precisely evaluated unless the skin envelope is returned back, so repeated manipulation of the volume and position of the graft may be needed, each time having the above mentioned risk of moving the skin flap back and forth.

Recently nonsurgical rhinoplasty using different filler materials have been popularized progressively. Obviating the need for operating room and general anesthesia and early and adjustable improvement of the deformity are the basis for this popularity. Even safe fillers such as hyaluronic acid have some reports of rare complications,^10^ but short term durability is the constant drawback of these fillers^11^^,^^12^ and usage of permanent fillers is limited due to the risk of adverse tissue reaction, infection and extrusion.^13^

Injectable autologous material that can be used beneath the nasal skin envelope will be very helpful to approach ideal surgical results. Autologous cartilage is the most frequent graft material in rhinoplasty and numerous studies have evaluated the long term viability of different forms of this valuable graft in rhinoplasty. Considering the consistency and shape of the cartilage that is highly dependent to the location of harvesting, multiple manipulations have been proposed to make it more malleable and advantageous for the purpose of camouflage. Crushed cartilage, minced cartilage and diced cartilage with or without wrapping in fascia are the most popular examples.

Various methods are introduced to crush the cartilage into desired consistency including cartilage morselizer, and cottle cartilage crusher. Crushed cartilage can be molded more easily and will have less palpable edges beneath the skin. It is easy to prepare and it is not time consuming. But the major problem is that crushing will cause a structural damage to the lacuna of the cartilage and will lead to loss of chondrocyte viability.^14^ Cackmack^14^ has well described a classification for the extent of crushing and has also evaluated the long term viability and permanence of each class in experimental studies. It is now evident that the more we crush a cartilage, the shorter it will survive as a graft.^14^ If a surgeon tends to crush the cartilage to an extent that becomes injectable, this will pose a great chance of future resorption and recurrence of the deformity.^14^

Dicing the cartilage block into small pieces with sharp blades is less harmful to the structure of the cartilage and its long term viability has been observed in multiple experimental and clinical studies.^15^ Diced cartilage if wrapped in fascia is a good material to augment the dorsum but it is usually more voluminous to be applied for correction of minor irregularities. If it is used without a cover of fascia, it is applicable as small volumes but it must be applied during the course of a rhinoplasty procedure, while there is no tight pocket of surrounding tissues to prevent its displacement.^15^

Besides, dicing or mincing the cartilage into desired small pieces that can pass from an injecting cannula using conventional blades is difficult and sometimes impossible. There are studies in the literature using otologic burr or other powered devices to mince the cartilage into injectable pieces, but the degree of damage to chondrocytes in the histologic evaluation may not support the possibility of long term survival.^16^

Preparing the injectable cartilage shaving is a simple procedure that does not demand powered devices or other uncommon facilities, it does not widely interfere with the integrity of the lacuna or chondrocytes, and in our experience it is comfortably applicable using 14 to 18 gauge needles or small diameter blunted tip cannulas. Vigorous crushing of the cartilage to make it injectable will destruct the anatomical structure of the lacuna and chondrocytes and will result in a more unpredictable course of resorption. Complementary procedures such as adding platelet rich plasma to the graft material can be performed to increase survival and regeneration.^17^

Cartilage shaving does have the valuable advantage of being injected through skin or mucosa after redraping the skin flap in cases of primary and secondary rhinoplasty, which enables the surgeon to correct the deformity incrementally and to the desired extent with precise evaluation of the surface changes. It can be also used without the need to elevate the skin flap and establishment of general anesthesia, as an outpatient procedure. In the latter situation, local and topical anesthesia can be used to harvest the conchal cartilage and also to inject the prepared cartilage shaving into the limited area of the nasal surface deformity.

The cartilage shaving may be also stored in the freezing (-19^o^c) temperature for 1 to 1.5 years before using in a touch up procedure. Long term clinical outcome in this study supports the finding that cartilage microstructure is not destroyed when prepared as injectable cartilage shaving, but more comprehensive studies on long term in vivo survival of this type of graft in animal models is being performed to prove its efficacy.

## CONFLICT OF INTEREST

The authors declare no conflict of interest.

## Note

Please cite this paper as:

Manafi A, Hamedi ZS, Manafi A, Rajabiani A, Rajaee AR, Manafi F. Injectable Cartilage Shaving: An Autologous and Long Lasting Filler Material for Correction of Minor Contour Deformities in Rhinoplasty. World J Plast Surg 2015;4(2):93-100.
